# Decision-Making at the Transition Period: Widening the Lens Around Psychosocial and Cultural Factors in Cleft Orthognathic Surgery

**DOI:** 10.1177/10556656261439955

**Published:** 2026-04-20

**Authors:** Holly Clegg, Kiki Mastroyannopoulou, Eliane Young

**Affiliations:** 1 Department of Clinical Psychology and Psychological Therapies, 12201 University of East Anglia Norwich Medical School, Norwich, UK; 2 4028 Cambridgeshire and Peterborough NHS Foundation Trust, Fulbourn, UK

**Keywords:** cleft lip and or/Palate, decision-making, orthognathic surgery, young people

## Abstract

**Objective:**

To explore what factors are considered by multidisciplinary team (MDT) professionals working in cleft lip and palate services when supporting young people in their decision-making for orthognathic surgery (OS) and how professionals understand and manage ethical dilemmas that arise.

**Design:**

A qualitative design was employed in which semistructured interviews were conducted remotely with NHS staff.

**Setting:**

Nine participants were interviewed across four NHS cleft services in the United Kingdom.

**Participants:**

The sample consisted of three Speech and Language Therapists, three Orthodontists, and three Surgeons involved in decision-making for OS.

**Results:**

Reflexive Thematic Analysis revealed three key themes and seven sub-themes:“navigating the decision-making process,” “Team-Centric,” and ‘Health Inequalities impacting on access to cleft services. Within these themes, professionals reflected on positions of power, the MDT environment, valued contribution of different professionals, external influences impacting on shared decision-making (SDM) and barriers to accessing care.

**Conclusions:**

The findings highlight the complex nature of balancing patient autonomy and beneficence during the decision-making process for OS while bringing awareness to factors that may pose a barrier to decision-making such as the presence of health inequalities, power dynamics and conditions of the MDT environment. The importance of optimizing the SDM environment and creating opportunities for liaison, particularly earlier in the pathway, are discussed. It is recommended that future research explores the impact of health inequalities on access to cleft care further, recognizing those that are currently disadvantaged in their treatment journey.

## Introduction

In the United Kingdon, patients born with a cleft lip and/or palate (CL/P) are commenced on a 20-year treatment pathway in which early surgery is required at around 3 to 6 months old to repair a cleft lip and between 6 and 12 months to repair a cleft palate.^1^ As patients progress along this pathway, there is often a transition of responsibility, whereby responsibility for decision-making transitions from the parent and/or caregiver to the young person. At the age of 16 (defined here as the “transition period”) young people experiencing adverse functional or aesthetic consequences may wish to elect for orthognathic surgery (OS). This procedure is usually performed once the young person has finished growing, involving re-aligning the jaws.^1^ It is often the first-time young people are positioned as primary decision makers with regard to their care.

The process of decision-making for OS is complex, requiring multi-disciplinary input and engagement in shared decision-making (SDM). As an outcome of Clinical Standards Advisory Group report published in 1998,^2^ specialist cleft teams in the UK became centralized. This was recommended with the aims of improving the standards of care, whereby multidisciplinary teams (MDTs) now provide care at regional cleft centres. Surgeons, forming part of the MDT, are required to discuss the advantages and risks to undergoing OS, with discussions often focusing on aesthetic and functional benefits.

As summarized by Ganoo and Sjöström,^3^ these often include observing an improved orthognathic and soft tissue profile, enhanced quality of life, and for some, improvements in speech. While postoperative outcomes are largely positive,^4^ risks of undergoing OS can include experiencing postoperative infection, blood loss, nerve injury, speech difficulties and relapse, with authors summarizing different aspects of these risks.^5–7^ While the need for OS is not limited to young people who have a CL/P, this cohort are of interest due to the longevity of treatments across their lifespan and with OS being offered at a point whereby responsibility for surgery decision-making often transitions to young people.

In addition to medical considerations, it is important to consider the psychosocial experiences of young people in the decision-making process for OS. Although evidence may suggest there is little difference between children and young people who have a CL/P compared to their unaffected peers with regards to psychological functioning,^8,9^ there is some evidence to suggest that children with a CL/P may experience increased anxiety and depression.^9^

Furthermore, a study by Stiernman et al^10^ which explored parents’ and healthcare professionals’ knowledge of psychosocial and educational outcomes affecting children with a CL/P, found several parents to report instances of their child being teased or receiving unwanted attention, with healthcare professionals raising concerns around low self-esteem, self-acceptance and coping with feelings of “difference.” Taken together, these findings highlight the importance of healthcare professionals having awareness of psychosocial factors when discussing treatment.

The rise of social media and impact on psychosocial functioning, should also be considered, in which Mironica et al,^11^ summarize literature on the association between taking and editing selfies, experiencing increased body dissatisfaction and consideration of cosmetic procedures. Stock et al,^12^ in interviewing healthcare professionals, further acknowledge the impact of social media platforms, reporting access to misinformation to be a barrier to delivering optimal care in cleft services.

With regards to addressing psychosocial factors, previous research reports that clinicians wish to address these factors but feel they do not have the relevant skills to meet patient's needs.^13^ Consistent with these findings is that none of the clinicians in Stiernman et al’^10^ study reported to have received formal training regarding psychological factors or adjustment to cleft, indicating further training may be beneficial. These findings are supported by Juggins et al,^14^ who found that over 80% of Consultant Orthodontists reported to have no formal teaching or training in psychological assessment and management emphasizing this training need further.

While discussing elective surgery, clinicians acknowledge best practice of engaging young people in SDM, which is both an essential and ethical obligation of craniofacial surgeons^15^ and extended MDT. While the adoption of SDM remains integral to care, research exploring the experiences of young adults in transitioning to adult cleft services, has suggested that this increased responsibility in decision-making can feel challenging, with young adults aware of the “permanency of results” if choosing to undergo elective surgery.^16^ Young adults in this study voiced that having a transition worker may have supported them in the decision-making process,^16^ however, despite NICE guidance recommending transition workers in transitioning to adult cleft services, it is acknowledged that teams are often positioned to manage transitions and young adults have been found to be unable to name or identify a professional undertaking this role.^17^

Additional dilemmas facing clinicians when engaging in SDM, include the patient, family and clinician holding different views around treatment and parental autonomy acting as a barrier to SDM.^18^ Furthermore, a study looking at young people's decision-making experiences around end-of-pathway cleft surgery discovered that once a malocclusion was pointed out to a young person, they questioned this aspect of their appearance, highlighting the importance of clinicians attending to their use of language during the decision-making process.^19^

In building on previous works that have explored decision-making experiences of young people and parents,^19,20^ this qualitative study adds to the literature by presenting the perspective of healthcare professionals, in exploring their understanding of psychosocial and cultural factors when supporting young people in their decision-making for OS.

## Method

### Design

Ethical approval was sought from the Faculty of Medicine and Health Sciences REC at the University of East Anglia and Health Research Authority. Local approvals were sought from research and development at the participating NHS sites.

Semistructured interviews were conducted with orthodontists, speech and language therapists (SLT's) and orthognathic surgeons involved in the decision-making process for OS, with a qualitative design being employed in allowing for in-depth exploration of clinician perspectives. Professional groups were selected based on the level of involvement each have in supporting young people in the decision-making process for OS, and with consideration of the chief investigator's (CI) professional role. Due to the nature of exploring psychosocial factors, and the CI's role as a trainee psychologist at the time of conducting the research, the decision was made to exclude clinical psychologists from recruitment, with consideration of the training and presumed knowledge this group possess in comparison to other professional groups. An interview topic guide was developed following consultation with a clinical Psychologist, orthodontist, surgeon and SLT working in the field and this was further role-played in ensuring that this felt appropriate for the setting.

The current study aimed to explore what factors are considered by different MDT professionals working in CL/P services when supporting young people in making decisions around OS, while also exploring how different professionals understand and manage ethical dilemmas that may arise during these interactions. The following research questions were addressed:

1. How do clinicians from different backgrounds experience and manage ethical dilemmas when supporting young people in making decisions about elective OS?
2. What factors do clinicians consider when consulting with young people about OS?
3. How does clinician awareness of psychosocial factors impact on the decision-making process when supporting young people in decision-making for OS?

## Procedure

Nine clinicians were recruited across four NHS cleft services. This sample size was decided upon in accordance with previous guidance by Braun and Clarke,^21^ which suggests recruiting 6 to 10 participants for small projects involving interviews. A recruitment email and participant information sheet were disseminated to clinicians in each team by the principal investigator. Following this, clinicians contacted the CI directly in expressing an interest in taking part in which they were followed up and consented into the study. The sample consisted of three SLT's, three surgeons and three orthodontists. Clinicians reported experience working in cleft services, ranged from three years to 20 + years.

Interviews were conducted remotely by the CI using Microsoft Teams, taking place from August to December 2023. The CI did not have any prior relationship with participants. A semistructured topic guide was implemented, with questions framed around communication and team working, perceived barriers and facilitators to engaging in SDM, professionals understanding and consideration of psychosocial and cultural factors during the decision-making process and management of ethical dilemmas. 90 min was allocated for each interview, which included time to answer questions, obtain consent, and debrief. Participants consented to the interviews being audio and video recorded and were informed how their data would be managed. Participants were assigned a participant ID and information was anonymized in maintaining confidentiality.

## Analysis

Three major themes and seven subthemes were formed through the process of conducting Reflexive Thematic Analysis (RTA).^22–25^ This approach was guided by the six-step process outlined by Braun and Clarke in 2006,^22^ but also included the researcher keeping a reflective diary throughout the process. In publishing a reflective commentary on RTA,^23^ and in their subsequent paper discussing quality practice,^25^ Braun and Clarke place emphasis on researchers’ reflecting on their theoretical assumptions and positionality. In accordance with these principles, the CI was reflective on their epidemiological stance, acknowledging the adoption of an interpretive constructivist approach. The CI was also aware of their role as a trainee clinical psychologist throughout the study and was mindful that this position may have influenced participant disclosure around psychological factors or narratives shared around the role of psychology within teams. In being aware of this, a reflective diary was kept in enhancing reflexivity and considering positionality and influence during the project. It is acknowledged that engaging with RTA, does not require input from a supervisory or research team,^25^ however for the purposes of the CI's doctoral project, of which this study was conducted, the supervisory team were consulted with the aims providing a reflective forum to discuss themes and constructing meaning from the data. In aiding this process, the CI kept a spreadsheet of codes and themes and developed a framework so that these could be discussed, however the CI acknowledges the potential contrast between employing coding frameworks and RTA as outlined by Braun and Clarke.^25^ The CI did not implement this framework as a way of achieving consensus, inter-rater reliability, or early identification of themes but alternately used this as a tool for reflection following all interviews being complete. A table representing the final themes is provided below (Table 1).

**Table 1.** Themes and Subthemes.

Theme	Subthemes
Theme 1: Navigating the decision-making process	*Finding a “balance”* *External factors impacting on the decision-making process.* *Managing positions of power* *The process of eliciting motivations for surgery*
Theme 2: “Team-centric”	*Opportunities to foster team communication.* *Valued contribution of* multidisciplinary team *(MDT) members*
Theme 3: Health Inequalities impacting on access to cleft care	*Equity and barriers to accessing care*

## Results

### Navigating the Decision-Making Process

Across interviews, it became apparent that many aspects of the decision-making process elicited feelings of discomfort for clinicians. Clinicians spoke of the challenges involved in balancing autonomy and beneficence, or when patients presented to clinic having been told OS was routine. The importance of understanding a young person's motivations for surgery was expressed, with clinicians voicing discomfort in being asked to operate when these reasons were not clear. This included exploring concerns that may be unvoiced such as those related to aesthetic or cosmetic reasons. Clinicians were also mindful that the presence of lots of professionals in one room, and layout of the room (eg, positioning of the dental chair) could create power dynamics and pose a barrier to SDM.

#### Finding a “Balance”

As clinicians spoke about the transition period, acknowledging the transfer of responsibility to young people, themes emerged around balancing patient autonomy and acting in the patient's best interest. Clinicians acknowledged the transition to SDM from previous medical models that may have seen professionals act in a more prescriptive manner, however despite this acknowledgement, clinicians were mindful of their role and responsibility in making decisions about surgery.

I’m constantly challenged, because of the nature of our work. It's a constant

challenge to try not to be to be paternalistic about things, but then there are some

times when you just have to say no and so finding the right balance. (P7).

Clinicians also shared their concerns around not wanting to make a situation “worse” and worrying if they had done the “right thing,” of which the desire for certainty was noted.

It's not my own ego, but it is hard because you're like, have I done the right thing

operating on her? and I, you know, she has good results. (P5)

What are the potential complications if I do something that leaves them worse off for something that isn't, you know, required to save their life? that's the responsibility that I have, or our team have. (P7)

I’m probably more risk averse than many of my younger colleagues would be, but I would rather wait until a patient is able to clearly say yes or no, rather than you

know, going ahead and say, well, let's just try it, let's just see how you go (P3).

On the theme of “finding a balance” clinicians also shared their experiences of supporting patients from different cultural and religious backgrounds. Below, a clinician shares their experience of working with a patient who identified as a Jehovah's Witness, in which an advocate was accessed in supporting them and the patient to understand each other's views around the risks involved in proceeding with OS.

We had a Jehovah's Witness advocate and that was very helpful in terms of helping me understand why it was important … because it's very easy to say, well, this is my belief, and I don't understand why you can do this to shift to … It's not about my belief, although you do have to acknowledge that your actions could impact on me … so there we did have a bit of a conversation about, but what does this mean to me? What does this mean to me if you were to die on the table? (P2)

In discussing working with a family from a Roma background, a clinician also refers to power dynamics and being positioned as an expert, sharing the following example:

There are some groups that still regard sort of anything a health professional says as “they are the health professional it's not my place to challenge it,” I actively encourage them to challenge it, but it doesn't sit comfortably with some groups. (P3).

Clinicians also expressed discomfort when young people presented to clinic having been told by their predecessors that they “need” OS or that OS is just the “next step” in the treatment pathway.

often you hear “well, I’ve just been told I need surgery” and I don't want to operate on someone who's only having this done because they've been told they need it. So, it's really important to me to kind of see what bothers the patients, what their concerns are and see if surgery is going to help. (P5)

The presentation of co-morbid mental health difficulties, and who holds responsibility was also highlighted as a concern with respect to “maintaining a balance” and keeping patients safe.

Severe depression, which is triggered by cleft, that's still severe depression that needs treating in mental health services not in cleft services. So sometimes there's a bit around … boundaries … where do we sit and that and how do we keep patients safe? (P2).

In discussing body-dysmorphia, the clinician also shared how it may be difficult to ascertain whether surgery will achieve the patients desired outcomes.

are you dealing with the body dysmorphia …? Probably. And it's harder to say that when you're doing cleft because there is actually a physical deformity … congenital deformity … but you do still get patients that have altered perception and if they have altered perception … is doing surgery actually going to give them a result that they will be happy with? (P2).

#### External Influences Impacting on Decision-Making

As families and young people increasingly seek out health information online, the pros and cons to accessing information online is debated. Clinicians acknowledged how online platforms may create a space for patients to come together, share narratives and ask questions, however concerns were raised around their regulation. Clinicians were also aware of the possibility of misinformation being shared, acknowledging the impact that social media platforms can have on young people's sense of self and appearance. Social media platforms such as TikTok and Instagram were reported to influence young people's perspectives on their appearance, bringing awareness to asymmetry and desire for surgery for aesthetic reasons.

Selfies aren’t too bad, but it's more when people take photographs of you, that's

when you see the asymmetry, because it's like you're looking at another person, not a mirror image…. So, photographs are double. They magnify the difference. (P2).

I wonder whether that's just, you know, the Instagram generation … the

people who are much more focused on faces than perhaps they ever have been.

Boys, girls alike … they often do want to go through treatments to average out their face to balance out their face. (P6).

Concerns were also raised around platforms being unmoderated, in which patients may be subject to misinformation, or may use online platforms to express dissatisfaction with their care or experiences with services and professionals.

Standard concerns about things being unmoderated, advice that we wouldn't

necessarily recommend is being shared quite readily without anybody there to say, oh no, no, don't do that. (P8)

they just kind of offloaded in this group and its sort of hard to read because

you can't then as a medical professional contact that parent and say, oh, I heard you were unhappy about this. Sorry about that, because there has to be those boundaries if they want to speak to us, to complain about it (P8).

In being aware of this, clinicians spoke of signposting patients to the British Orthodontic Society and patient experience videos in accessing reliable and up-to-date information.

We do try to stop patients accessing… you know, non-sanitized … Internet

publications of whatever sort. We try to say, listen, if you're gonna read anything

online, make sure it's sort of British stuff, Orthodontic Society cleft world stuff so that you're as sure as you can be that you're getting what we would be considered as our treatment modalities rather than what might be happening overseas or somewhere else. (P6)

Although concerns were raised around seeking out health information online, patient communities and social media platforms were also noted as being helpful in terms of patients hearing peer experiences of undergoing OS.

it's good to have some other agency or some other group that speak to the patients or inform the patients about that which is useful in terms of they're getting a view from some other forum. I know one or two patients who've … who've got their information from TikTok … they followed someone who is, who has shown their experiences on TikTok, of having had the surgery done. (P4).

#### The Process of Eliciting Motivations for Surgery

Psychological safety is highlighted as a central component in patients feeling able to disclose their concerns during SDM, of which clinicians were aware that this may involve “picking out” motivations for surgery.

Under all that, there are reasons why people probably would like to have the benefits of having jaw surgery. But I think it's really important to kind of pick that out … You always wanted to get it out of the patient. Why they … why they want surgery. (P5).

Another clinician also describes this process as “deep diving” into the “layers” of concern.

Really deep diving into … the burden that these patients carry and the sort of layers of not dissatisfaction but the layers of concern related to appearance and how they’re perceived and how many of those are things that we can improve and how those are related to things that we look at and go, “I could do you the best bite and the best jaw surgery, but I sense you're still not going to be entirely happy with things,” and it's about sort of riding out those decisions. (P3).

One of the reasons clinicians felt patients may be more hesitant to share their concerns, is anticipating that patients feel it is not acceptable to proceed with surgery for aesthetic or cosmetic reasons, with gender differences also being acknowledged with regard to pragmatism, awareness of emotions and expectations around surgery.

There is a perception within the NHS that you can't really ask for things for an

aesthetic basis. It's all got to be functionally related … (P3).

You know, it's a cosmetic thing, why should I have access to cosmetic treatment? (P2)

they've boxed it because as a bloke they shouldn't be going down that pathway of

caring what their nose looks like, of being vain, whereas the girls handle it a lot more. They're just much more with it. They're just more. “Yeah. Don't like my nose, I want something done about it”, “I have the right to want something done about it, why shouldn't I have a good nose?” You know., so they’re … in some ways … much more emotionally in-tune and pragmatic about what they want than the boys are, the boy's pragmatism boxes them in. (P2).

Asking open questions and using language that does not imply positionality towards surgery, was highlighted as an important factor in supporting young people to make decisions and avoiding creating feelings of difference or dissatisfaction with appearance.

You could have someone who's quite happy and then you tell them … that they're not right. So … you're creating a problem and … that is challenging … What language do you use? rather than saying right/wrong, you might say “your top teeth are behind your bottom teeth, are you happy with that appearance? Can you function OK?” not, it is wrong, and moving away from that concept of they as a patient are wrong in any way or not normal (P2).

These concerns were further highlighted in the following quote.

I'm really cognizant of not creating … that awareness that, “oh, maybe I look different”, “Is there something wrong with me?” So, I can distinctly remember seeing a young girl … she had no issues with how she looked. She had brilliant confidence … and I'm very cognizant that I don't want to create something in her mind. (P5)

In exploring young people's understanding and motivations for surgery, clinicians also spoke about the role of psychology within the MDT.

but having the psychologist on board has been utterly invaluable because they've

been able to sort of dissect out where the areas of concern are rather than …

I'm unhappy with everything, they've been able to dissect that … what elements that are specifically related to your cleft are you unhappy with? what elements like to talk about with the surgeons? What elements are you worried about with your speaking? What elements do you not like about your teeth? and they can break it down. And so, the patient comes back sort of prepared with more specific questions (P3).

The role of psychologists was also raised with respect to supporting clients with autism or learning needs and understanding capacity.

now and again you do you get a patient with special needs, with perhaps ASD … and then picking apart their cognition of what they're letting themselves in for, understanding their capacity for decision-making … that is that is a real challenge but that's a really small subset of our patient group. Certainly, those guys I think we would ask the psychologist to help (P6).

#### Managing Positions of Power

While trying to elicit motivations for surgery and engage young people in SDM, clinicians reflected on both the advantages and potential drawbacks of coming together with other professionals and the patient in one room. Professionals acknowledged how meeting as a team with the patient present provided helpful opportunities to have discussions with colleagues and for patients to observe that the decision-making process requires significant thought and care. In contrast, professionals reflected on how the presence of lots of professionals may feel “intimidating” and “overwhelming,” resulting in patients being less likely to disclose motivations for surgery. It was also raised that the “modern day MDT experience” may bring back memories for clients of earlier challenging times in their childhood.

we do get returning adults who perhaps had a challenging experience as kids and

they find that perhaps the MDT environment is a bit overwhelming despite the fact

actually … they may see the psychologist beforehand and they prep

them for what a … modern day MDT experience is like, but still they say, listen, I can't cope with that … that brings me back to my childhood … which was

challenging (P6).

If you have, you know, like three consultant surgeons and a couple of trainees and a

consultant orthodontist like, you're not going to bare your soul and be like, oh,

actually, I'm really conscious about this, you'd just be like, I'm here to talk about

surgery, so I think taking the patient out of that environment and having a you know, less intimidating chat is really valuable (P5)

The presence and positioning of the dental chair was also highlighted in relation to creating power dynamics between professionals and the patient, of which clinicians spoke about the particular placement of the chair behind a wall and importance of having this positioned at eye level with patients.

So those clinics do happen with them sitting down and everyone else towering

above, and I wonder if that can have a negative impact or a positive one, not you

know, the sort of feeling lower down than everyone because they're sat down, but

the fact that everything is in the same room and everyone's hearing the same thing. (P9)

and a dental chair which is behind the kind of half high wall so the patients don't walk in and see a dental chair, they come in and … we sit them down and … we make sure it's that sort of … subtle things, but we make sure that we're always at eye level with them, so that the seats that the patient sit on are slightly higher than ours. So that when we are looking, we're on an equal level and we space it to make it sort of as informal as possible. (P3)

While the drawbacks of the MDT environment are acknowledged, conversely, the MDT environment was felt to provide opportunities for discussion and for everyone to hear the same thing.

We have everyone in the room at the same time. Now the advantage of that is we

can have around the table discussions where we can thrash out all these things.

(P7)

I think it's useful for them to see that we have really thought about it, and we've had a proper a discussion about it on the other side of things. (P7).

While holding both the advantages and challenges to meeting as an MDT in mind, clinicians were mindful of the need to create psychological safety.

It's gonna be about psychological safety isn't it, if they don't know you … why

they’re going to discuss it? They don't know how it's gonna land. And they've made … sometimes they’ll have made assumptions. (P2).

The following quote illustrates the importance of patients having a safe space and creating psychological safety, in being able to understand the information provided and feeling able to ask questions.

And you know, the patients came back and said to [clinician] why are they feeding me wafers … if I'm going to be asleep during an operation … it's not like a pink wafer, what they're talking about is a bit of plastic that fits between your teeth, and I've gone. “oh,” and the fact that they haven't felt sort of comfortable enough to ask that question reveals quite a lot about how the conversation goes, and you know, there is so much that we probably think we're doing right, but without asking the patients, we don't know. (P3).

### Theme 2: “Team-Centric”

Clinicians reflected on the nature of having centralized and localized teams, reflecting on opportunities for liaison and how this was influenced and differed by team set up. Some clinicians felt they had many opportunities to liaise with their colleagues, speaking of the advantages of being able to have “ad hoc” discussions. Other teams desired more opportunities for team liaison and reported that there were not many opportunities, resulting in delays to updating patient-facing information and discussing feedback. While clinicians reflected on the structure of services, discussions evolved around equity, such as the availability of different professionals and provision of services at each site, having access to regular dental care, and factors that may pose a barrier to accessing care, such as travel costs and child-care considerations.

#### Opportunities to Foster Team Communication

Clinicians commented on the advantages of being able to have “ad hoc” conversations and liaise with their colleagues, whereas other teams relied more heavily on the support of community-based colleagues and connecting remotely.

the key to a strong and functional team is that you do have the opportunities just to have corridor conversations and that you do bump into your colleagues. (P6)

very dispersed team in that we rely really heavily on the support of our community-based colleagues. We've never had the funding all centralised, so we have very good links across primary and secondary care and that's really, really important in terms of the way lots of things we do are run (P1).

Other clinicians felt there could be more opportunities for liaison in-between clinics, emphasizing the importance of having opportunities to meet as a team.

You know, we don't really and actually it would probably be good if we did, because there are a few things that we need to think about and so like, we have a leaflet that we think needs updating and that's almost a year and nothing has happened and probably because we don't have any platform for meeting up in between those clinics. (P9)

I should make more time to try and set up a way of discussing people …  patients … 

difficult patients with other colleagues … because of the very specialized nature of it … you have to go out of region to find someone who really knows what you're talking about … that's one of the things that that I would like to develop (P7)

#### Valued Contribution of MDT Members

Clinicians highlighted the significance of building relationships with patients across the treatment pathway, of which the orthodontists role was particularly highlighted due to the level of interaction they often have with families and young people.

the orthodontist having potentially known the child … or the patient rather, since they were a child, often may have … quite an insight into that patients life, their thoughts their, you know their personality and so sometimes the orthodontist can … can take a bit more of a lead (P6).

SDM is also noted within teams, of which a clinician describes the nature of the MDT as “everybody is a clinic lead.”

I'd very much hate to work in a team where, for example, the surgeon led everything and was, you know, was seen as the clinical lead when everybody's a clinical lead because everybody's part of the service and … input to the care …  (P6).

Although the value of each speciality within the MDT is noted, themes arose around how SLT's view their role, and how the role of SLT's are viewed in relation to the decision-making process.

I think we have quite an important role to play … in different ways … I think we have an important role to play clinically, in terms of our specialism and in terms of advising. (P1).

if it was to go in order of a contribution and impact on that patient's journey, then I

think probably we would maybe come bottom of the pile. I … feel like perhaps our psychology team and orthodontists have even more involvement with those patients than we do. (P6)

Speech and language therapy colleagues tend to do the assessment and just feed in and give some general advice around risk to speech to the patients … and that's

about it. They don't have a huge impact otherwise … and not very much direct

influence on treatment planning or what we do with the surgery. (P4).

### Theme 3: The Impact of Health Inequalities on Access to Cleft Care

While reflecting on the nature of centralized and de-centralized teams, clinicians were mindful of the various barriers patients may face in receiving care. Clinicians raised issues relating to equity across services, financial and travel implications, and the provision of services in different regions.

####  Equity and Barriers to Accessing Support

In speaking about health equalities and the perceived impact on access to services, a clinician highlighted the requirements placed on patients precluding attending clinic. In particular, the clinician highlighted the expectation that young people will be able to access a dentist regularly in promoting “good oral health,” which may impact on being able to attend clinic.

I don’t see the same mixture of backgrounds in our cleft orthognathic than I would expect…. That may be because there are so many other factors that preclude them actually turning up to clinic. You know, you think of all the hurdles that we put in place. They’ve got to be able to attend appointments … they’ve got to be able to see a dentist on a regular basis, to have good oral health. They’ve got to be able to engage with the communication from the hospital from the admin team, inviting them to appointments, and I would say all those things … exclude quite a significant proportion of people that we look after (P3).

Consideration is also given to childcare, financial and employment implications that may impact on access to healthcare, highlighting the various barriers that may exist, prior to surgery decision-making.

And we select patients to be seen there by postcode and so many of them have said “there's no way I’d have got up to the (clinic location). You know, I can't do it with picking up the other kids” … or even the bus … even though transport costs are reimbursed on the day … It's down to having that amount of disposable income that you can pay out bus fares for a parent and child. (P3)

maybe that's through lack of … opportunity to access our care. You know, I

think probably families who are in those situations that, they need to be at work, for example, and they're their ability to get away from work, to bring their kid to

treatment. It is definitely much more of a challenge for some than others. (P6).

This was also discussed in the context of the transition period, and how receiving treatment may impact on education and employment in addition to impacting financially on the young person if being required to travel.

When you're a teenager, often they look at it as, “oh, I'm going to miss a session of school every six weeks, that's not such a bad thing” but when you are putting yourself under pressure for A Levels or going to university or starting an apprenticeship, and you're looking at whether your employer is going to let you out of work for such regular times … or how you're going to get there independently and the cost of travel, there are lots of different factors on somebody's desire to do all of that as a pre requirement to the jaw surgery. (P3).

In hearing from clinicians who work across different regions, it was acknowledged that processes may also vary between sites, which may include the advice and guidance provided and availability of professionals in clinics. In reflecting on equity across services, clinicians raised the following points:

The other is a lack of consistency in approach across the whole of the [region]. So, there might be inequity in terms of that service because they may not hear the same thing from my local, you know like from my colleagues who do the clinic in (different locations). (P4).

There's a bit of a mixed economy in that regard. Some of the clinics I will go to or my colleague who also does orthognathic surgery, goes to … there will be other

clinics that there is no maxillofacial orthognathic surgeon attending. So those

patients would most likely have to travel to [other site location] to be seen by the

surgeons to get the advice about what they think is possible. (P7).

## Discussion

The findings offer insight into the experiences of clinicians when supporting young people in their decision-making for OS, of which three main themes were identified through conducting RTA^22–25^: “Navigating the decision-making process,” “Team-centric” and “Health inequalities impacting on access to cleft care.”

Clinician narratives revealed a shared experience of internal conflict, particularly for those positioned with the decision to offer or not offer surgery. Clinicians described the challenging nature of balancing patient autonomy and beneficence, expressing concern around “doing the right thing.” This was underpinned by a desire for certainty in the decision being made, with clinicians experiencing discomfort when positioned as expert or when young people presented for OS having been told they need surgery.

Previous research exploring the decision-making experiences of young people suggests they feel “tied” to the treatment pathway, therefore “going along” with surgery as this is viewed as being routine.^19^ The literature supports the narratives of clinicians in this study, in that young people may present to surgery feeling that this is the next step in their care, creating feelings of discomfort. The desire of clinicians to promote SDM and for young people to express a degree of certainty around their motivations for surgery, however, sits in contrast to the experiences of young people, who may feel that taking this position is burdensome and therefore they wish to delegate this responsibility.

With the adoption of SDM acknowledged, it should be considered that in some contexts, clinicians adopting decision-making roles may have a function in reducing uncertainty and unpredictability.^26^ This reinforces clinician narratives around balancing patient autonomy and beneficence. In responding to this dilemma, various factors were found to mitigate against feelings of discomfort. Feeling held and contained within the MDT and accommodating “ad hoc” discussions and opportunities for liaison were felt to facilitate the decision-making process. Similarly, clinicians felt the input from Clinical Psychologists was particularly beneficial in trying to understand motivations for surgery, or when questions arose around a young person's capacity. This echo's findings from Stock et al,^12^ in which healthcare professionals have raised the importance of collaborative working, and valuing input from MDT colleagues in providing optimal cleft services.

With regards to exploring psychosocial factors, previous research suggests that clinicians do not feel they have the right skills to address psychosocial factors with their patients.^13^ Consistent with this, clinicians in the study highlighted how the SDM environment, where the patient is joined by multidisciplinary professionals in one room, can be intimidating and may pose a barrier to SDM. This has also been reported by young people in sharing their experiences of decision-making.^19^

Clinicians acknowledge that patients may experience dissatisfaction with their appearance, underpinning motivations for surgery, however this can sometimes be difficult for clinicians to elicit due to the nature of the decision-making environment. Consequently, it is often the case that patients share information in their individual consultations with clinicians, highlighting the need to consider power dynamics and promote psychological safety in eliciting motivations and reaching collaborative decisions. Clinicians spoke of reducing the number of professionals in the room and trying to mitigate power dynamics by making modifications to the environment that promote SDM. Furthermore, having regular opportunities to liaise and seek support from colleagues may enable SDM and reduce feelings of discomfort, particularly for those who are positioned to decide about offering surgery. It may also be beneficial, to foster opportunities for liaison earlier on in the treatment pathway, in paying consideration to the transition period and experiences of young people at this life stage.

Socioeconomic factors have been reported as a barrier to accessing healthcare, in which financial implications associated with travelling to appointments have been found to impact attendance.^27^ Consistent with this, clinician narratives highlighted several barriers to accessing care, including the need for patients to travel to different sites and the associated financial, education and employment considerations that this involves. Although de-centralized clinics may offer opportunities to access care closer to home, clinicians voiced inequity between services, describing this as a “mixed economy,” which may contribute to health inequalities.

Clinicians were mindful that patients may experience co-morbid mental health difficulties and were conscious of not proceeding with OS when this was unlikely to meet the patients’ expectations. Social media platforms were noted as a significant influence on patients’ perception of themselves and their appearance and during the SDM discussions clinicians were aware of not biasing the young person's decision or creating feelings of dissatisfaction in their use of language. Themes arising from clinician interviews, were consistent with what is reported about the influence of social media platforms in the literature. Clinicians remarked on the impact of social media platforms on accentuating “feelings of difference,” which echo's findings from the review by Mironica et al^11^ surrounding access to social media, perception of body image and consideration of surgery.

Clinician awareness and consideration of cultural factors differed across professionals, with some sharing that these considerations had not arose in their work and could be considered more by the team. In contrast, where cultural factors were acknowledged, a clinician referred to having an advocate present in managing decisional conflict and another reflected on being positioned as expert. Although the present study brings awareness to health inequalities that may preclude patients from accessing services, as this was not the focus of the study, this arguably provides a limited account of these experiences. As noted by professionals, this is a topic that understandably warrants further research and allocation of resources. Developing a greater understanding of the factors that may preclude patients accessing care, and increasing awareness of health inequalities, will allow services to consider how these may be addressed. Further research may also wish to explore how teams consider and incorporate SDM models at different stages of the treatment pathway, of which it would be of benefit to include a greater representation of cleft centres from different regions, and wider professional groups, in research. While the findings contribute to our understanding of psychosocial factors, particularly among professionals supporting children and young people receiving care for a CL/P at the transition period, the authors acknowledge limitations in their methodology, and reference Braun and Clarke more recent guidance on conducting RTA and adhering to best practice.^22–25^

## Data Availability

Fully anonymized data are not publicly available due to participant confidentiality and consent restrictions.
